# Development of a specific MPXV antigen detection immunodiagnostic assay

**DOI:** 10.3389/fmicb.2023.1243523

**Published:** 2023-09-07

**Authors:** Ian Davis, Jackie M. Payne, Victoria L. Olguin, Madison P. Sanders, Tamara Clements, Christopher P. Stefan, Janice A. Williams, Jay W. Hooper, John W. Huggins, Eric M. Mucker, Keersten M. Ricks

**Affiliations:** ^1^Diagnostic Systems Division, United States Army Medical Research Institute of Diseases, Frederick, MD, United States; ^2^Pathology Division, United States Army Medical Research Institute of Diseases, Frederick, MD, United States; ^3^Virology Division, United States Army Medical Research Institute of Diseases, Frederick, MD, United States

**Keywords:** mpox, immunofluorescence, antigen assay, clade-specific, Magpix

## Abstract

Human monkeypox (mpox) has recently become a global public health emergency; however, assays that detect mpox infection are not widely available, largely due to cross-reactivity within the *Orthopoxvirus* genus. Immunoassay development was largely confined to researchers who focus on biothreats and endemic areas (Central and West Africa) until the 2022 outbreak. As was noted in the COVID-19 pandemic, antigen detection assays, integrated with molecular assays, are necessary to help curb the spread of disease. Antigen-detecting immunoassays offer the advantage of providing results ranging from within min to h and in lateral flow formats; they can be deployed for point-of-care, home, or field use. This study reports the development of an mpox-specific antigen detection immunoassay developed on a multiplexed, magnetic-bead-based platform utilizing reagents from all research sectors (commercial, academic, and governmental). Two semi-quantitative assays were developed in parallel and standardized with infectious mpox virus (MPXV) cell culture fluid and MPXV-positive non-human primate (NHP) sera samples. These assays could detect viral antigens in serum, were highly specific toward MPXV as compared to other infectious orthopoxviruses (vaccinia virus, cowpox virus, and camelpox virus), and exhibited a correlation with quantitative PCR results from an NHP study. Access to a toolbox of assays for mpox detection will be key for identifying cases and ensuring proper treatment, as MPXV is currently a global traveler.

## 1. Introduction

**Figure 1:**
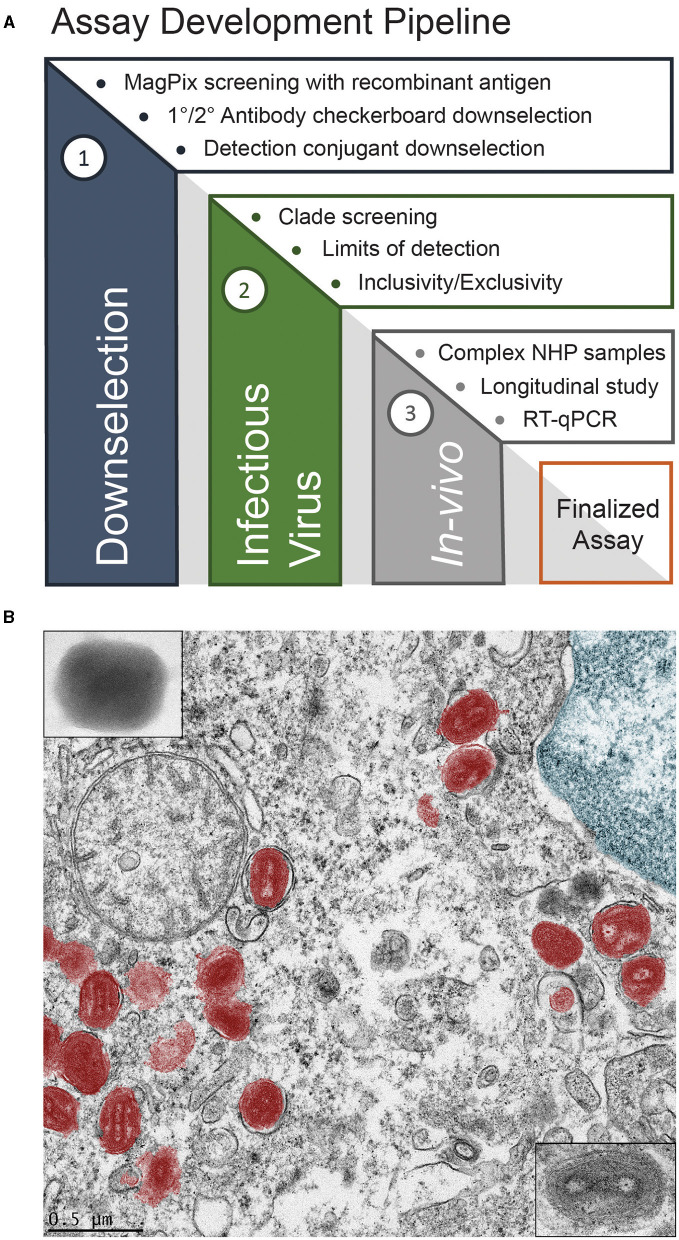
Assay development **(A)**, and false color transmission electron microscope (TEM) image of MPXV in a liver cell **(B)**.

Human monkeypox (mpox), caused by the mpox virus (MPXV), recently became a global health emergency (Hirani et al., 2022). This emergency status was driven by mpox outbreaks occurring in dozens of countries in 2022, including those in Europe and the Americas, where mpox was not endemic. As a member of the *Orthopoxvirus* genus, mpox phenotypically presents similarly to smallpox, which is caused by the variola major virus (McCollum and Damon, 2014). The recent outbreak, combined with the similar presentation to smallpox, necessitates the development of new mpox-specific diagnostic tools for both public health and military force health protection. Both endeavors would benefit from a rapid diagnostic device that could be deployed far forward, either at the point-of-care or in the field. The key features of such a device would be its robustness, ease of usage, and its ability to differentiate between MPXV, vaccinia, cowpox, and variola major to rule out potential, incredibly lethal smallpox infection or infection due to vaccinia virus (Henderson and Arita, 2014). To work toward such a device, we employed the assay development pipeline shown in Figure 1A. This pipeline begins by down-selecting relevant antibodies using recombinant antigens, screening against infectious viruses, and verification through *in vivo* models, as compared with other analytical techniques.

MPXV, shown in false color in Figure 1B, was discovered in 1958 in Denmark from an outbreak in laboratory primates (Magnus et al., 1959). The first documented human cases were found in 1970 in the Democratic Republic of the Congo, Liberia, Sierra Leone, and Nigeria during efforts to eradicate smallpox (Foster et al., 1972; Ladnyj et al., 1972). The first outbreak outside of Africa occurred in 2003 in the midwestern United States due to the importation of infected rodents from Ghana (Ligon, 2004; Reed et al., 2004). The recent global outbreak was unique in that high rates of human-to-human transmission via intimate, sexual contact was common (Heskin et al., 2022; Karagoz et al., 2023). The current gold standard for diagnosing mpox is polymerase chain reaction (PCR) to detect viral DNA (Li et al., 2006; Brown and Leggat, 2016; Maksyutov et al., 2016; Davi et al., 2019; Altindis et al., 2022). However, PCR requires careful sample handling and extraction; it also requires sophisticated instrumentation that may only exist in centralized labs and usually takes hours to days to produce a result. As such, there is a need for an assay that can provide immediate results to clinicians and commanders to inform their decision-making. Lateral flow antigen detection assays, which were ubiquitous during the COVID-19 pandemic, fit this requirement as they are accessible, easy to use, require minimal sample preparation, and offer a rapid response time (~15–30 min). Prior to manufacturing these lateral flow devices, immunoassay development typically begins with more conventional, ELISA-style instrumentation.

We utilized a multiplexable, magnetic-bead-based Luminex system, the Magpix^®^, for our immunoassay development. This is because assay development workflows require smaller amounts of reagents and lower sample volumes, all while affording increased signal-to-noise over traditional 96-well plate ELISAs; they also offer multiplexing capability for more rapid down-selection. Conserving time and resources is critical to rapid assay development prior to transitioning to more point-of-care formats. Typically, two antibodies are needed to design an antigen-detection immunoassay. A primary antibody is attached to magnetic beads to capture the antigen of interest, and a secondary antibody is used for detection, as depicted in Supplementary Figure S1. A library of monoclonal antibodies (MAbs) that react with antigens from the vaccinia virus (VACV) and other orthopoxviruses antigens have been developed at the United States Army Medical Research Institute of Infectious Diseases (USAMRIID) over the years to support orthopox vaccine development efforts. However, those antibodies lacked specificity to a particular orthopoxvirus. An MAb, 69-126-3-7, α-A29, was found to bind the MPXV protein, A29, but not the homologous protein from other orthopoxviruses (Hughes et al., 2014). This MAb was acquired and used as the primary (1°) MAb to develop a Magpix assay for specifically detecting MPXV A29. Institutional and commercial MAbs were screened for use as a secondary (2°) MAb to complete the antibody sandwich. Top-performing antibody pairs were verified using serum samples of cynomolgus macaques positive for MPXV from a previous study (unpublished).

Smallpox was one of the worst diseases in human history, so much so that a multi-decade global effort was undertaken to eradicate it. Routine vaccination against smallpox provided protection against mpox; however, the practice ended in the 1980s. Due to the lack of vaccination, the global population becomes more susceptible to poxviruses every year. As such, the natural spread of MPXV, or other similar poxviruses filling the ecological niche, and the potential threat of bioterrorism with smallpox are growing concerns. Therefore, there is a critical need to develop modern diagnostics for poxviruses, both to support public health by limiting the spread of mpox and to prevent the reemergence of smallpox.

## 2. Materials and methods

### 2.1. Reagents

Recombinantly expressed vaccinia virus (VACV) A27L was purchased from AlphaVax Inc. (Research Triangle Park, NC, USA), and Clade II MPXV A29L was purchased from Sino Biological Inc. (Chesterbrook, PA, USA). Clade I MPXV A29L was recombinantly expressed and purified in a previous study (Heraud et al., 2006). Commercial α-A29 MAbs, namely, clone IDs 31, 27, 32, 25, and 17, were purchased from Sino Biological Inc. (Chesterbrook, PA, USA). The previously identified α-MPXV A29 MAb (69-126-3-7) was acquired from the Center for Disease Control (CDC) (Hughes et al., 2014), and the USAMRIID α-VACV A27 MAb (4B4) was acquired from a previous study (Hooper et al., 2009). Magnetic microspheres and an xMAP^®^ antibody coupling kit were purchased from Luminex Inc. (Austin, TX, USA). Streptavidin, R-phycoerythrin conjugate (SAPE) was purchased from Life Technologies Corporation (Eugene, OR, USA), the PE/R-Phycoerythrin conjugation kit—Lighting-Link^®^ was purchased from Abcam (Cambridge, UK), and the EZ-link™ Sulfo-NHS-LC-Biotin, No-Weigh™ Format kit was purchased from Thermo Fisher Scientific (Waltham, MA, USA). Phosphate buffered saline, Tween-20, and skim milk powder were purchased from Sigma-Aldrich (St. Louis, MO, USA).

### 2.2. Microsphere preparation

Antibodies were covalently linked to microspheres following the manufacturer's instructions. For all steps that required removing solutions, the microspheres were immobilized by placing a rare-earth magnet adjacent to the reaction vessel, and the solution was removed using a pipette. The microspheres were protected from light during the incubation steps with aluminum foil. Briefly, 12.5 million microspheres were washed three times with 500 μL of activation buffer and resuspended in 274.5 μL of activation buffer. Next, 144.0 μL of sulfo-N-hydroxysulfosuccinimide and 81.5 μL of 1-ethyl-3-(3-dimethylaminopropyl) carbodiimide hydrochloride solutions were added, and the tubes were gently rotated for 20 min. After activation, the microspheres were washed three times with coupling buffer, and the antibody was added at 4 μg per million microspheres. The reaction was allowed to incubate for 2 h, after which the microspheres were washed three times with 500 μL of PBS-T (phosphate buffered saline with 0.05% Tween-20), resuspended at 12.5 million microspheres per mL in PBS-T, and stored at 4°C.

### 2.3. Antibody conjugation

Antibodies were covalently coupled with biotin and phycoerythrin (PE) using commercial kits and following the manufacturers' instructions. Biotinylation of MAbs was achieved using the EZ-link™ Sulfo-NHS-LC-Biotin, No-Weigh™ Format kit. Briefly, 50–60 μg of MAbs were made to react with a 20-fold molar excess of freshly prepared sulfo-NHS-LC-biotin (sulfosuccinimidyl-6-[biotin-amido]hexanoate) at room temperature for 30 min. After the reaction, excess biotin was removed by dialyzing against PBS-T. Antibody-PE conjugates were generated with the PE/R-Phycoerythrin conjugation kit—Lighting-Link^®^. Briefly, 6 μL of a modifier reagent was added to 60 μg of MAb (1.0 mg/mL), before being added to lyophilized R-phycoerythrin. The mixture was allowed to react in the dark overnight. After the reaction, 6 μL of a quencher reagent was added, and the conjugates were used without further purification.

### 2.4. General assay procedure

Assays were developed on the Magpix^®^ platform using white, Costar, round-bottom 96-well plates. The plates were loaded with 2,500 microspheres per well, placed on a magnetic block for 1 min, and manually decanted. Samples were diluted in 5% skim milk in PBS-T (SM), and 50 μL was applied to each well. The plates were then covered and allowed to incubate with 450 rpm shaking for 1 h. After incubation, the microspheres in each well were washed three times with 100 μL of PBS-T. Secondary antibody conjugates (PE or biotin-labeled) were diluted to 1 μg/mL in SM, and 50 μL was added to the appropriate wells. The plates were then covered and allowed to incubate with 450 rpm shaking for another hour. After incubation, the microspheres were washed three times with PBS-T. For plates using 2°-PE conjugates, the microspheres were suspended in 100 μL of PBS-T and read using the Magpix^®^ instrument. For plates using 2°-biotin conjugates, SAPE was diluted to 10 μg/mL in SM, and 50 μL was added per well before covering and incubating with shaking at 450 rpm for 30 min. After the final incubation, the microspheres were washed three times with PBS-T, suspended in 100 μL of PBS-T, and read using the Magpix^®^ instrument. A pictorial representation is provided in Supplementary Figure S1.

### 2.5. Test samples

Whole blood samples were derived from a therapeutics study performed in cynomolgus macaques under the Good Laboratory Practices standard described by the Food and Drug Administration. Briefly, animals were intravenously infected with the Zaire strain of MPXV (Clade I), as previously described (Mucker et al., 2022a,b). To assess the efficacy of ST-246 at the time of lesion onset, animals were orally treated with one of two concentrations (10 mg/kg or 20 mg/kg) of ST-246 or placebo, starting at 72 h or 96 h post-exposure. At 72 h, 50% of the placebo and 7 of 12 ST-246 treated animals had observable lesions, whereas 100% of all animals had observable lesions at 96 h. Ethylenediaminetetraacetic acid (EDTA)-treated whole blood samples were collected on the day before the challenge, on the day of the challenge, every 3 days after the challenge, and upon death. The samples were processed to sera for analysis. All treated animals survived the exposure, whereas all placebo-treated animals succumbed to infection.

### 2.6. Extraction and qPCR

Nucleic acid was extracted from the samples using Qiagen's QIAamp DNA Mini Kit. A quantitative, pan-orthopox assay was used to detect the poxvirus hemagglutinin (HA) gene. Assays were performed and analyzed on a LightCycler 2.0 or similar. The limit of quantitation was 50 copies per 5 μL reaction (10,000 genomes/mL). This procedure has been validated and described elsewhere (Mucker et al., 2017).

## 3. Results

### 3.1. Screening antibody combinations for VACV A27 and MPXV A29 detection

**Figure 2:**
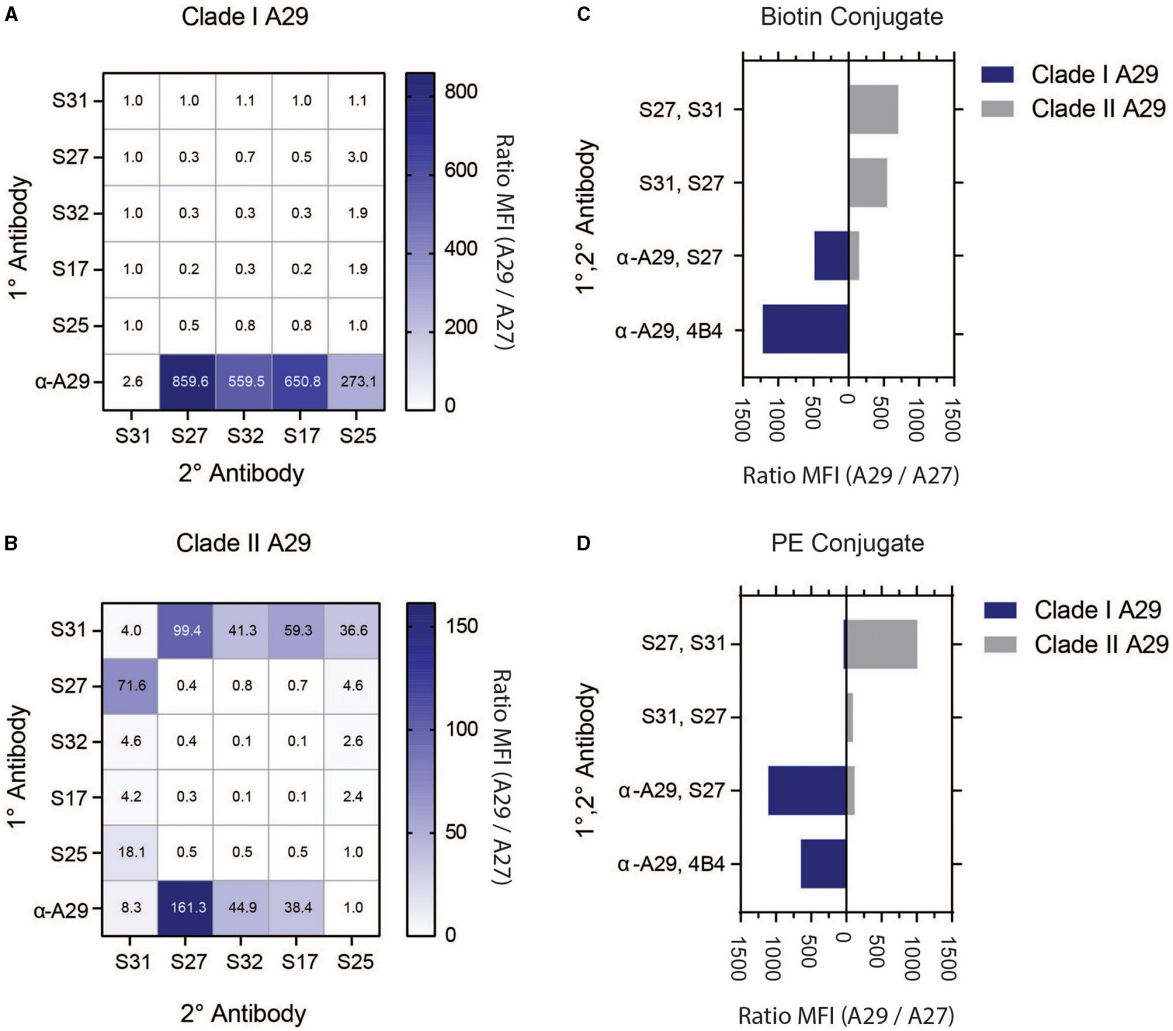
Screening of MAbs and detection strategy with recombinant antigens. The 1° MAbs (vertical axis) are covalently linked to magnetic microspheres. 2° MAbs (horizontal axis) are biotinylated, and detection is achieved with SAPE. Clade I MPXV A29 is shown in **(A)**, and Clade II MPXV A29 is shown in **(B)**. Clade selectivity of down-selected MAb pairs using 2°-biotin conjugate, as shown in **(C)**, and 2°-PE conjugate, as shown in **(D)**. Data are presented as signals using MPXV A29 divided by signals using VACV A27.

The USAMRIID has researched orthopoxviruses for many decades and has a wide inventory of monoclonal antibodies (MAb) against vaccinia virus (VACV), but the majority of these antibodies are highly cross-reactive among the genus (Edghill-Smith et al., 2005; Kitamoto et al., 2005; Heraud et al., 2006; Hooper et al., 2007, 2009; Su et al., 2007; Golden and Hooper, 2008, 2010; Golden et al., 2011, 2012; Mucker et al., 2013, 2017, 2018, 2020, 2022a,b; Pittman et al., 2015; Kota et al., 2023; Taha et al., 2023). A previous study by the Center for Disease Control (CDC) discovered MAb 69-126-3-7 (α-A29) that binds MPXV protein A29 (ortholog of VACV A27) with high specificity (Hughes et al., 2014). The CDC and USAMRIID further characterized this monoclonal antibody. At the beginning of assay development, this was the only monoclonal in the peer-reviewed literature that was shown to be specific to MPXV. As such, we targeted MPXV A29 for MPXV-specific immunoassay detection. We acquired MAb 69-126-3-7 from the CDC, five commercial A29 MAbs (S17, S25, S27, S31, and S32), and USAMRIID's MAb 4B4, which was previously used to detect VACV A27 (Hooper et al., 2009). To screen for viable antibody pairs, each of the MPXV A29 MAbs was covalently coupled to magnetic microspheres to serve as capture MAbs (1°) and covalently linked with biotin to serve as detector MAbs (2°). SAPE was used as a fluorescent reporter. Antibody pairs were screened against recombinant Clade I and II MPXV A29, as well as VACV A27, at 100 ng/mL in a checkerboard fashion to determine the best antibody pairs (Figures 2A, B, Supplementary Figure S2). All commercial MAbs were cross-reactive with Clade I MPXV A29 and VACV A27 when used as a capture antibody. When α-A29 was used as the capture antibody, there was notable one-way reactivity with MPXV A29 with all commercial MAbs as detectors, except for S31. For Clade II MPXV A29, one-way reactivity was found for several pairs that utilized α-A29 or S31. The most promising pairs were α-A29/S27, S31/S27, and S27/S31. Based on these results, the aforementioned pairs were down-selected for further assay optimization.

### 3.2. Selection of a labeling strategy

Selected pairs from the initial screening, as well as MAb 4B4 from previous research (Hooper et al., 2009), were directly labeled with phycoerythrin (PE). Direct PE conjugation of the 2° MAbs simplifies assays by eliminating the need for a third assay incubation with SAPE, and it can improve the overall assay performance. As stated above, MAb pairs were screened against Clade I and II MPXV A29, as well as VACV A27 (100 ng/mL). The signal was expressed as the MPXV A29 response divided by the VACV A27 response to demonstrate specificity (Figures 2C, D) or as median fluorescence intensity (MFI) (Supplementary Figure S3). α-A29 was screened as 1° with either 4B4 or S27 as 2°, S31 was screened as 1° with S27 as 2°, and S27 was screened as 1° with S31 as 2°. In addition to showing one-way reactivity toward MPXV, α-A29/4B4 was also specific to Clade I MPXV A29 with biotin (4B4-B) or PE (4B4-PE) conjugation. The α-A29/S27 pair reacted to both MPXV A29 clades, whether biotinylated (S27-B) or as a PE conjugate (S27-PE), with S27-PE generating more signal for Clade I MPXV A29. S27/S31 and S31/S27 were both selective for Clade II MPXV A29, with S27/S31-PE generating the highest signal. The MAb pairs were further characterized using infectious viruses, *vide infra*.

### 3.3. Down-selection using infectious viruses

After screening numerous MAb pairs and reporter-labeling strategies against recombinant antigens, we proceeded to test the assays against infectious viruses. Various orthopoxviruses (MPXV Zaire '79, MPXV Katakombe, MPXV US 2003, MPXV current, VACV Lister, VACV IHDJ, VACV Western Reserve, cowpox Brighton, cowpox wild-type, and camelpox Somalia) were previously cultured in Vero cells, and cell slurries were harvested for antigen detection. Relative virus concentrations were determined through plaque assay to be 10^6^-10^8^ plaque-forming units per milliliter (pfu/mL). Two-fold dilutions of the cell slurries were screened with the selected MAb pairs, and the results are shown as signal divided by negative slurry (Table 1). Among the MAb pairs screened, only pairs that used α-A29 as 1° MAb, yielded an appreciable signal over the uninfected cell slurry. As a 2° MAb, S27 performed similarly for both clades and with either label (biotin or PE), whereas 4B4 maintained Clade I selectivity when biotinylated but lost selectivity and response when PE-conjugated. Based on the reactivity and signal-to-noise ratio, three MAb pairs were selected for further development: α-A29/4B4-B, α-A29/S27-B, and α-A29/S27-PE.

**Table 1:** Antigen detection with infectious virus.

	α**-A29**^**a**^				**S31** ^**a**^		**S27** ^**a**^	
	**4B4** ^b^		**S27** ^b^		**S27** ^b^		**S31** ^b^	
	**2**°**-biotin**	**2**°**-PE**	**2**°**-biotin**	**2**°**-PE**	**2**°**-biotin**	**2**°**-PE**	**2**°**-biotin**	**2**°**-PE**
MPVX Zaire ′79	1706.6	405.4	701.5	845.4	1.1	0.9	1.0	0.0
MPXV Katakombe	1916.7	436.2	792.8	920.0	1.2	0.9	1.0	0.0
MPXV US 2003	1.1	219.3	829.5	807.0	2.7	1.5	1.0	0.0
MPXV current	1.1	458.0	904.1	774.6	6.2	1.1	1.0	0.0
VACV lister	39.1	33.7	38.3	23.6	1.3	0.4	1.0	0.0
VACV IHDJ	17.5	14.2	14.9	10.6	1.2	0.9	1.0	0.0
VACV WR	32.9	27.1	49.9	26.7	1.5	0.6	1.0	0.0
Cowpox BR	46.8	37.1	53.4	34.6	1.5	0.6	1.0	0.0
Cowpox wt	11.4	11.7	13.2	10.4	1.1	0.9	1.0	0.0
Camelpox Somalia	1.0	0.8	1.2	1.1	1.0	0.9	1.0	0.1

^a^The 1° MAb is covalently linked to magnetic microspheres.

^b^The 2° MAb is biotinylated (detection with SAPE) or directly PE-labeled.

### 3.4. Determination of the limits of detection

**Figure 3:**
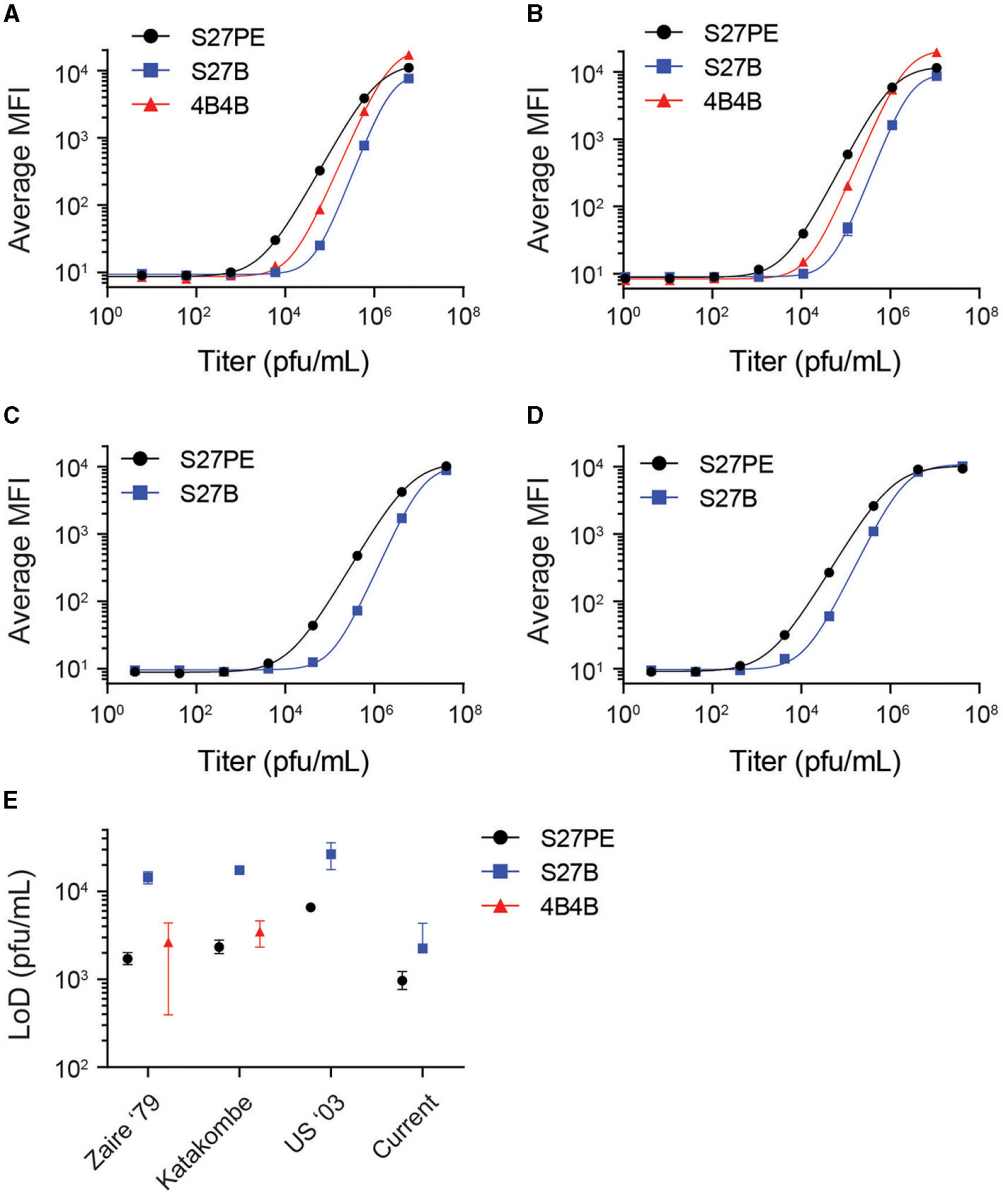
Limits of detection with infectious virus. α-A29 is used as the capture MAb, with S27 or 4B4 as the 2° MAb. S27-PE, S27-B, and 4B4-B are depicted as black circles, blue squares, and red triangles, respectively. Median fluorescence intensity (MFI) is shown as a function of virus titer in pfu/mL for MPXV strains Zaire '79 **(A)**, Katakombe **(B)**, US 2003 **(C)**, and the currently circulating strain **(D)**. Data were fit with a 4-parameter sigmoidal function. Interpolated limits of detection of the antigen assays are shown with 90% confidence intervals in **(E)**. The 4B4 assay does not detect Clade II MPXV and is excluded from **(C, D)**.

Three antibody pairs were tested against infectious MPXV strains to determine their limits of detection (LoD). α-A29 was used as the 1° MAb, with 4B4-B, S27-B, or S27-PE as 2° MAb. Slurries of infectious virus were initially diluted at a 1:2 ratio in the assay buffer prior to 10-fold serial dilutions (Figure 3). The data were fit with a four-parameter sigmoidal function, and LoDs were interpolated from the fitted function using three standard deviations greater than the average of a blank titration as a cutoff value (Figure 3E, Supplementary Table S1). For Clade I strains, the LoD for S27-PE and 4B4-B were both in the low thousands of pfu/mL, while S27-B was ten-fold less sensitive. For all strains, S27-PE had a much lower LoD than S27-B.

### 3.5. MPXV assay verification with longitudinal animal model samples

**Figure 4:**
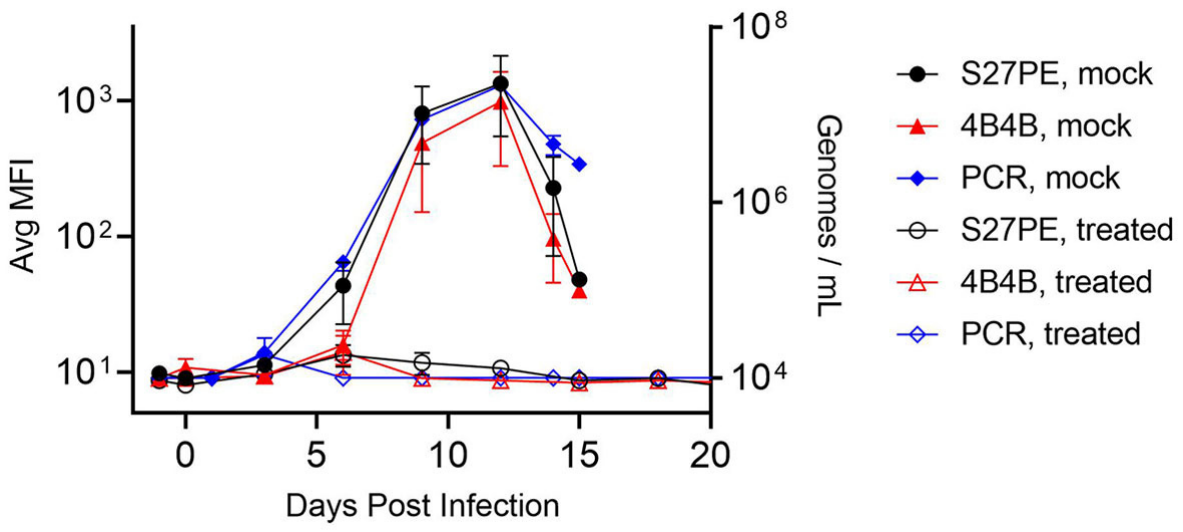
Detection of MPXV with specific assays. NHPs were challenged on day 0 and treated with ST-246 (hollow) or placebo (filled) at 72 h. Serum was assayed using α-A29 as the 1° MAb, with S27-PE (black circles) or 4B4-B (red triangles) as the 2° MAb. qPCR data are shown as blue diamonds. Error bars are SEM for 3 treated or 4 placebo NHPs.

Two assays, α-A29/S27-PE and α-A29/4B4-B as 1°/2°, were carried forward to test for antigenic reactivity with serum samples from an institutional, legacy MPXV animal study. Non-human primates (NHPs and cynomolgus macaques) were given an intravenous challenge of MPXV Zaire '79 (day 0) and treated with tecovirimat (ST-246) (3 NHPs, 20 mg/kg) or placebo (4 NHPs) 72 h later. Placebo NHPs experienced severe illness and succumbed between days 13 and 15, while all treated NHPs survived. Serum was collected on the day before the challenge, on the day of the challenge, every 3 days after the challenge, and during terminal bleeds for NHPs that succumbed on day 14. Serum samples were diluted 1:20 and tested for MPXV A29 (Figure 4). Legions appeared for all NHPs on days 3 and 4, and the placebo NHPs showed detectable MPXV A29 in the serum beginning on day 6 and peaking on day 12. Both assays detected MPXV A29 in the serum from challenged, but not treated, NHPs, even though the treated NHPs developed lesions. There was no statistical difference between the signal from the S27-PE assay and that from the 4B4-B assay in terms of both total signal and S/N. Samples were also screened for the presence of MPXV DNA using qPCR, the current “gold-standard” for detection in this model (Mucker et al., 2017). The antigen detection assays were highly correlated with the qPCR results (Pearson r > 0.98, Supplementary Figure S3).

## 4. Discussion

Diagnostic tools that are sensitive, specific, accurate, and accessible are invaluable to correctly identifying the etiology and ultimately stopping widespread disease, in the public health sector and for military force health protection. PCR became a household term during the COVID-19 pandemic, as this nucleic acid-based test was the only FDA-cleared method for determining SARS-CoV-2 positivity during the first year of the pandemic. Once rapid antigen tests became widely available, infected individuals were better able to self-diagnose and isolate without having to wait 24–48 h for test results (Gronvall, 2021). Molecular diagnostic assays, such as PCR, are much more sensitive and specific than immunodiagnostic assays. Primer and probe design for specific nucleic acid targets coupled with signal amplification contributes to the exquisite sensitivity of these assays. Immunodiagnostic assays, such as ELISA and lateral flow assays, are not as sensitive as molecular diagnostic assays, such as PCR, but they are often much faster and require far fewer sample processing steps (e.g., nucleic acid extraction) and consumables.

The world was still in slow recovery from the COVID-19 pandemic when mpox cases began to rise at an alarming rate in non-endemic areas in early 2022. Due to the ongoing development of orthopoxvirus countermeasures, potential vaccines, as well as PCR assays, were already available for diagnosing patients among naïve populations (Li et al., 2006; Maksyutov et al., 2016; Mucker et al., 2017; Papadakis et al., 2023). There were no commercially available antigen assays for MPXV or any orthopoxvirus outside of lab-derived tests at research institutions. Prior to the 2022 outbreak, the commercial market for antibodies or recombinant proteins for MPXV or other orthopoxviruses was limited. Orthopoxviruses are large DNA viruses with genomes that encode approximately 200 proteins. The large number of proteins complicates the development of antigen-detection immunoassays because biologically relevant targets must be carefully selected (Shchelkunov et al., 2002). The Diagnostic Systems Division at USAMRIID has developed several research-grade assays using monoclonal antibodies developed by the Virology Division, but these reagents lack antigenic specificity, leading to “pan-orthopoxvirus” immunoassays. Research on orthopoxviruses over the years from the USAMRIID, the CDC, and other academic and governmental institutions have highlighted protein targets of interest for MPXV-specific detection, including the α-A29 MAb discovered in 2014. The recent outbreak invigorated interest from commercial sources to develop MPXV-specific MAbs against A29 as well. Upon testing these MAb sources with our USAMRIID orthopox MAb collection, we successfully designed and verified an MPXV-specific MPXV A29 detection assay. The assay was verified against well-characterized, longitudinal NHP samples from an MPXV therapeutics study. We could detect MPXV A29 in the untreated animals but observed no signal from the treated animals, and the results showed an excellent correlation with qPCR data. The signal became detectable on day 6 in the untreated cohort and persisted until the end of the study. While this assay cannot be considered an indication of a correlate of protection, it was certainly interesting to observe the absence of a signal in the treated cohort.

Our aim at the beginning of this study was to create an MPXV-specific immunoassay. We did not expect to find clade-specific reactivity. MPXV A29 from the two clades differ by only two amino acids, the substitutions are relatively conservative, histidine to arginine and arginine to histidine, and they are not located on the heparin-binding domain that confers specificity to α-A29 (Hughes et al., 2014). Further modeling is necessary to assess the binding sites, antibody kinetics, and epitope binning to understand this phenomenon and determine whether it is due to the antibody itself or the labeling chemistry. The most plausible explanation is that the relatively small size of biotin, as compared to PE, allows it to access an attachment site that competes for a binding epitope necessary for identifying Clade II A29 but not Clade I A29. This development effort also highlights the risks associated with relying on recombinant antigens for assay development. Several commercial MAb pairs produced promising results during the initial screening, but none of them were effective when assayed against infectious viruses. MAbs developed using recombinant antigens may not translate to clinically relevant assays, demonstrating the need to use infectious viruses in antibody discovery pipelines.

## 5. Conclusion

Two antibody pairs that detected MPXV with high sensitivity and selectivity were identified. The LoDs for the assays were in the low thousands of pfu/mL. The developed assays were verified with serum samples from an animal model of mpox. Future research will focus on testing the assay on known MPXV-infected human samples, as well as other matrices of clinical relevance to orthopoxviruses (pustules, scabs, and swabs). Additionally, we plan to transition these assays to a lateral-flow format so that they can be deployed far forward for point-of-care or field use. Accurate, specific, and timely diagnostics are key to mission readiness. As such, our diagnostic toolbox needs to be well-stocked to prepare for the next outbreak or pandemic.

## Data availability statement

The original contributions presented in the study are included in the article/Supplementary material, further inquiries can be directed to the corresponding author.

## Ethics statement

The animal study was approved by the United States Army Medical Research Institute of Infectious Diseases IACUC. The study was conducted in accordance with the local legislation and institutional requirements.

## Author contributions

ID and KR conceived and designed the study and wrote the manuscript with input from all coauthors. ID, JP, VO, and TC conducted the experiments. CS provided advice. JW provided the TEM image. JHo provided reagents and advice. JHu and EM conducted the NHP study. All authors contributed to the article and approved the submitted version.
