# Supplementary material for: Development of a specific MPXV antigen detection immunodiagnostic assay
Source: Front Microbiol. 2023 Sep 7;14:1243523. doi: 10.3389/fmicb.2023.1243523 (PMC10516133; doi:10.3389/fmicb.2023.1243523)
Supplement: Supplementary file 1 [file Data_Sheet_1.docx]

Supplementary Material

Development of a specific MPXV Antigen Detection Immunodiagnostic Assay for differentiating Orthopoxviruses

Ian Davis^1^, Jackie Payne^1^, Victoria Olguin^1^, Madison Sanders^1^, Tamara Clements^1^, Christopher P. Stefan^1^, Janice A. Williams^3^, Jay W. Hooper^2^, John W. Huggins^2^, Eric M. Mucker^2^, Keersten M. Ricks^1^*

*** Correspondence:** Keersten M. Ricks: [keersten.m.ricks.civ@health.mil](mailto:keersten.m.ricks.civ@health.mil)

^1^Diagnostic Systems Division, United States Army Medical Research Institute of Diseases, Fort Detrick, Maryland, USA

^2^Virology Division, United States Army Medical Research Institute of Diseases, Fort Detrick, Maryland, USA

^3^Pathology Division, United States Army Medical Research Institute of Diseases, Fort Detrick, Maryland, USA

# Supplementary Figures


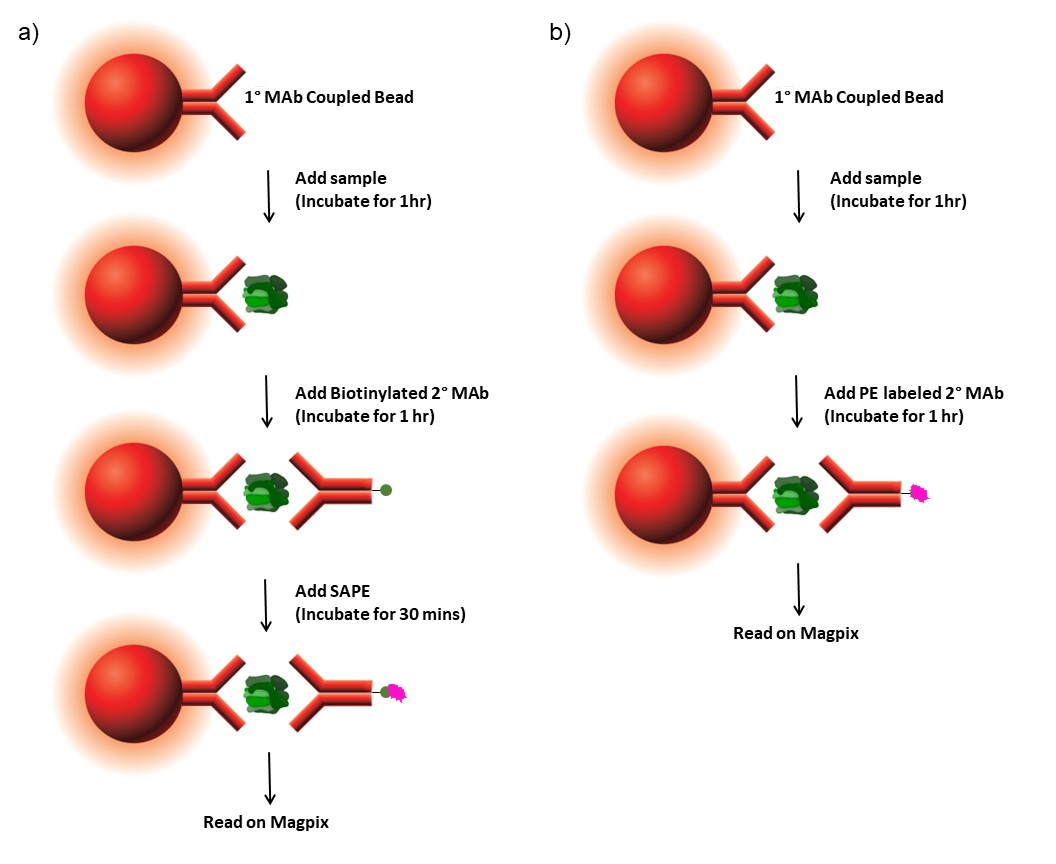


**Supplementary figure 1.** Schematic representation of a Magpix antigen sandwich assay. 1° MAbs are covalently linked to fluorescently labeled magnetic microspheres. The microspheres are incubated with sample and allowed to capture the antigen of interest, if present. The microspheres are then incubated with 2° MAbs to recognize the antigen. 2° MAbs are covalently labeled with either biotin, a, or phycoerythrin (PE), b. If the 2° MAb is labeled with biotin, an additional incubation with streptavidin-phycoerythrin (SAPE) conjugate is required. Signal is read by the Magpix instrument as the median fluorescence intensity generated by phycoerythrin per bead.

**Supplementary figure 2.** Initial screening of MAbs with recombinant antigens. 1° MAbs (vertical axis) are covalently linked to magnetic microspheres, 2° MAbs (horizontal axis) are biotinylated, and detection is achieved with SAPE. Data are presented as signal divided by negative control and colored with a heat map. Clade I and II MPXV A29 are shown in a, and b, respectively, and VACV A27 is shown in c.

**Supplementary figure 3.** Biotinylation vs direct PE labeling of 2° MAbs with recombinant antigens. The 1° MAbs (vertical axis) are covalently linked to magnetic microspheres, 2° MAbs (horizontal axis) are biotinylated (detection with SAPE) or directly PE labelled. Data are presented as median fluorescence intensity (MFI) and colored with a heat map.

**Supplementary figure 4.** Correlation matrix for mpox assays measuring longitudinal samples from NHPs challenged MPXV. Pearson r values are calculated for data presented in Figure 6. Correlation coefficients are colored with a heat map.

# Supplementary Tables

**Supplementary table 1.** Limits of Detection for MPXV specific assays.

| MPXV | 2° Antibody^a^ | | |
| --- | --- | --- | --- |
| Strain | S27-PE | S27-B | 4B4-B |
| Zaire '79 | 1712.0 | 14364.4 | 2633.2 |
| Katakombe | 2326.0 | 17425.5 | 3481.7 |
|  |  |  |  |
| US '03 | 6594.4 | 26524.0 | ND^b^ |
| Current | 964.9 | 2238.9 | ND^b^ |

^a^LoD values are given in pfu/mL.

^b^Not determined
